# Parental Perspectives on Psychiatric Comorbidity in Preschoolers With Autism Spectrum Disorders Receiving Publicly Funded Mental Health Services

**DOI:** 10.3389/fpsyt.2019.00107

**Published:** 2019-03-12

**Authors:** Filippo Muratori, Marco Turi, Margherita Prosperi, Antonio Narzisi, Giovanni Valeri, Silvia Guerrera, Elisa Santocchi, Fabio Apicella, Caterina Lattarulo, Sara Calderoni, Stefano Vicari

**Affiliations:** ^1^Department of Developmental Neuroscience, IRCCS Fondazione Stella Maris, Pisa, Italy; ^2^Department of Clinical and Experimental Medicine, University of Pisa, Pisa, Italy; ^3^Fondazione Stella Maris Mediterraneo, Potenza, Italy; ^4^Child and Adolescence Neuropsychiatry Unit, Department of Neuroscience, Children Hospital Bambino Gesù, Rome, Italy

**Keywords:** child behavior checklist 1.5–5, affective problems, anxiety problems, young children, oppositional problems, ADHD problems, multicomorbidity, sleep problems

## Abstract

An increased prevalence of psychiatric comorbidity (PC) in individuals with Autism Spectrum Disorders (ASD) is consistently reported. While several studies have examined PC in school-aged children, adolescents and adults with ASD, investigations on PC in preschoolers are less common. In this study, we explore the prevalence and the type of PC in a sample of 989 preschoolers with ASD through the DSM-Oriented Scales (DOS) of the Child Behavior Checklist (CBCL 1½-5) and their possible links with the core features of ASD and cognitive functioning. Results indicated that 37.8% of the sample had at least one PC in addition to ASD; these subjects displayed significantly higher Total score (*p* = 0.02) and Social Affect score (*p* = 0.003) on the ADOS-based calibrated severity scores (CSS), as well as lower (*p* ≤ 0.0001) performance IQ (pIQ) compared to ASD individuals without PC. As far as the specific DOS, Affective Problems (AP) were detected in 23.4% of the whole sample, ADHD Problems (ADHD) in 17.3%, Anxiety Problems (AXP) in 16.7%, and Oppositional Problems (OP) in 7.9%. These different comorbidities were isolated in 195 subjects (Mono-comorbid group: 19.7% of the whole sample), while 179 subjects (18.1% of the whole sample) had two or more types of PC (Multi-comorbid group). One-way ANOVA revealed that subjects with multi-comorbidity have statistically significant lower pIQ and higher Total score and Social Affect score on CSS-ADOS. Specific differences for each type of comorbidity and gender differences were also discussed. Taken together, results indicate a considerable presence of PC in preschoolers with ASD that should be accurately considered during the assessment and diagnosis process in order to plan a tailored intervention based not only on core symptoms of ASD, but also on comorbid psychiatric condition since preschool age.

## Introduction

Autism Spectrum Disorders (ASD) are neurodevelopmental disorders characterized by persistent social communication difficulties as well as restricted interests, repetitive activities and sensory abnormalities (1). Substantial heterogeneity exists in ASD in terms of genetic susceptibility (2), neural underpinnings (3), clinical presentation (4), medical and psychiatric comorbidities (5), response to treatment (6), and developmental trajectories (7). In particular, studies consistently reported an increased prevalence of psychiatric comorbidities (PC) in individuals with ASD compared with typically developing (TD) controls (8–10). The type and the prevalence rate of PC in ASD considerably vary across studies, according to the demographic and clinical features of patients (e.g., sex, age, core symptom severity, intellective functioning) as well as the assessment modalities (11). PC of children with ASD predicts poorer prognosis (12), and are associated with psychological distress in their parents—see the recent systematic review and meta-analysis (13). It is worth mentioning that the presentation of PC in the ASD population could be different than PC in the general population. Therefore, there is a considerable risk for mis– or under–diagnosis of PC (and consequently under–treatment) if symptoms are presumed to be part of ASD (i.e., diagnostic overshadowing) (14). Vice versa, there is also the possibility that the co–occurrence of psychiatric disorders may mask or obscure the core symptoms of ASD and thus contribute to difficulties of accurate and timely diagnosis of ASD (15).

Moreover, developmental characteristics such as age, intellectual functioning, and socio–communicative abilities may interfere on the presentation and expression of PC in individuals with ASD. In particular, difficulties in communication are intrinsically part of the ASD features and could impact–especially if there is an associated intellectual disability– on the ability of patients to express their own emotional and behavioral problems, and this is particularly true in the preschool years.

While several studies have examined PC in school–aged children (16), adolescents (17), and adults (18–20) with ASD, investigations on the presence of PC in preschoolers with ASD are less common. Importantly, several studies demonstrated a high presence of multiple PC in ASD children and adolescents. For example, Simonoff et al. (21) used structured assessments in a sample of 255 ASD children aged 10–14 years detecting that 41% had two or more co–occurring disorders and more than a third had three or more disorders in addition to ASD. Specifically, Social Anxiety Disorder (29%), Attention–Deficit/Hyperactivity Disorder (28%), and Oppositional Defiant Disorder (28%) were the most common PC; while the prevalence of Major Depressive Disorder (0.9%), Dysthymic Disorder (0.5%), and Conduct Disorder (3%) appeared minimal. Also, Leyfer et al. (9) implemented a modified version of the Kiddie Schedule for Affective Disorders (K–SADS) in a sample of participants aged 5–17 years and found that the majority of their sample had at least two PC in addition to ASD. However, the authors suggested a likely underestimation of the diagnosis of multiple comorbidity resulting from the methodology adopted. A high multiple PC was subsequently confirmed in other ASD samples of children and adolescents with ASD (22–27).

The difficulty to find reliable and accurate diagnostic tools to detect PC in preschoolers with ASD could contribute to the relatively sparse studies in this area (27). Among instruments used to measure comorbid psychopathology in young children with ASD, the Child Behavior Checklist (CBCL) was considered robust in their measurement properties –see Hanratty et al. (28) for a recent systematic review on this topic. In fact, the CBCL's syndrome scales demonstrated good instrument quality and validity (29, 30), and the CBCL's DSM–Oriented Scales (DOS) showed similitudes in psychometric properties with regard to consistency, reliability and cross–informant agreement (31, 32). In addition, previous studies have shown that DOS are valid for discriminating related DSM–diagnoses in participants both in the CBCL 6–18 (33–35) and in the CBCL 1.5–5 (36). The use of checklist measures allowed the clinicians to highlight a strong *continuum* between preschool behavioral and emotional problems and psychopathology in later childhood (37–40) and even adulthood (41). In addition, it has been shown similitudes in psychopathology between preschoolers and older children and adolescents with a high concordance between parental report at early age and the following direct evaluations of the same participants at an older age (42). The use of the same clinical CBCL thresholds in both school children and preschoolers is under discussion, since the applying of lower threshold scores in preschoolers has proved useful (43). In fact, a tendency of parents to underestimate affective and atypical reactions in preschoolers as compared to older children emerges, in particular for depressed symptoms (44) and disruptive behaviors (45).

Some previous studies used the CBCL 1½−5 to investigate the PC of preschoolers with ASD. Hartley et al. (46) evaluated 169 young children with ASD and found that about one third (34.3%) of the sample had a Total Problems score in the clinically significant range, while the most frequent clinically significant scores in syndrome scales were Withdrawn (70.4%), Attention (38.5%) and Aggression (22.5%), with a high degree of comorbidity. Hartley and Sikora (47) examined coexisting emotional and behavior problems in a sample of 157 boys and 42 girls with ASD aged 1.5–3.9 years. Results indicated that female toddlers exhibited more sleep and affective problems than matched males. Tseng et al. (48) identified more severe internalizing problems and higher scores in Withdrawn, Social Problems, Thought Problems, and Attention Problems scale in ASD toddlers than in typically developing children; moreover, 73.1% of the patient sample—composed of 67 ASD preschoolers—had at least one CBCL syndrome scale score in the clinically significant range, while 47.7% had two or more. Giovagnoli et al. (49) reported significantly higher rates of behavioral and emotional problems in children with ASD as compared to their TD peers: specifically, in all the three broadband scales (total, internalizing, and externalizing problems), and in all syndrome scales, with exception of Somatic Complaints and Sleep Problems. Vaillancourt et al. (50) conducted a longitudinal investigation across four time points of children with ASD aged 3 to 6 years and detected that internalizing and externalizing behaviors co–occurred at high rates across time, and, on average, declined slightly over time. However, high/stable course of internalizing or externalizing problems were found in a considerable part of the sample (23.2 and 13.5%, respectively).

While the abovementioned investigations used the broadband and the syndrome scales of the CBCL 1½−5, in the present paper we preferred the DOS to investigate PC in a more precise way. The same method was applied in a recent study on the prevalence of Anxiety Problems and Attention Deficit Hyperactivity Disorder Problems in a sample of preschool and early elementary aged children with ASD (51). Compared to this study we widened the number of PC investigated, describing the presence and the type of four PC (Affective Problems, Anxiety Problems, Attention Deficit Hyperactivity Disorder Problems and Oppositional Problems) through the DOS of the CBCL 1½−5–Parent Report Form in a much larger sample of ASD preschoolers. Possible correlations with demographic and clinical variables (gender, intellective functioning, core ASD features) were also evaluated.

## Methods

### Participants

The sample (Table 1) included 989 preschoolers with ASD between 16 and 75 months of age (mean age: 44.0 months; *SD*: 13.8 months) recruited by three different Italian care centers for children: specifically, 498 children from IRCCS Fondazione Stella Maris in Pisa, 323 children from Bambin Gesù Children's Hospital in Rome, and 168 children from Stella Maris Mediterraneo Foundation in Matera. These children were selected among individuals who received a diagnosis of ASD according to DSM−5 criteria (52), or of autistic disorder, Asperger disorder, and pervasive developmental disorder–not otherwise specified according to DSM–IV criteria (53), performed by a multidisciplinary team including a senior child psychiatrist and an experienced clinically trained research child psychologist.

**Table 1:** Demographic, clinical characteristics, CBCL broad–band, and DSM–IV Oriented scales scores in the total sample (*n* = 989).

		**Total sample (*n* = 989)**
Gender (Male/Female)		820 (83%):169 (17%)
		**Mean (SD); Range**
Age (months)		44.01 (13.76); 16–75.15
Performance IQ		79.21 (23.30); 30–138
ADOS Calibrated Severity Score—Social Affect		6.11 (1.96); 1–10
ADOS Calibrated Severity Score—RRB		6.95 (0.06); 1–10
ADOS Calibrated Severity Score—Total score		6.26 (0.06); 3–10
**CBCL**	**T–Score Mean (SD); Range**	**Number (%) of subjects with CBCL score in the borderline or clinical range**
CBCL—Total problems	58.40 (10.87); 50–94	363 (36.7)
CBCL—Internalizing problems	60.13 (10.27); 50–93	479 (48.4)
CBCL—Externalizing problems	54.90 (9.62); 50–97	220 (22.2)
CBCL—Pervasive developmental problems	68.64 (9.71); 50–98	682 (69)
**CBCL DSM–IV oriented scales**	**T–Score Mean (SD); Range**	**Number (%) of subjects with CBCL score in the borderline or clinical range**
CBCL—Affective problems	58.82 (8.78); 50–95	231 (23.4)
CBCL—Anxiety problems	56.74 (8.13); 50–100	165 (16.7)
CBCL—Attention Deficit/Hyperactivity problems	57.80 (6.97); 50–76	171 (17.3)
CBCL—Oppositional problems	54.52 (6.09); 50–80	78 (7.9)

*Number of subjects with CBCL score in the borderline or clinical range are reported*.

Clinical diagnosis was confirmed by ADOS, the gold–standard standardized interviewer–rated measure for child observation and assessment of skills in communication, social interaction, quality of play and imagination. In this study, ADOS–G (54) and ADOS−2 (55) were applied. According to two already published algorithms (56, 57), the Calibrated Severity Score (CSS) was obtained for each participant based on ADOS Total score and sub–scores Social Affect (SA) and Restricted Repetitive Behaviors (RRB). CSS range is 1–10 and it allows comparing different versions and modules of ADOS. Moreover, the CSS provides a measure of autism symptoms that is independent of age and language ability and thus is better suited than the ADOS scores for assessing the severity of ASD (58). The scores of ADOS–G were previously converted in ADOS−2 scores (SA, RRB and Total score) on the basis of the new algorithm proposed by Gotham et al. algorithm (59). The total and the CSS domains were calculated for Toddler Module of ADOS−2 on the basis of Esler et al. (60) to facilitate a direct comparison to other modules of ADOS−2.

As far as cognitive evaluation, a number of standardized tests were used to assess intellectual abilities due to differences in the verbal skills and functioning level of children. These included: the Leiter International Performance Scale–Revised (LIPS– R) (61), the Griffiths Mental Developmental Scales–Extended–Revised (GMDS–ER) (62), and the Italian version of Wechsler Preschool and Primary Scale of Intelligence (WPPSI) (63). When the tool provides a mental age (MA), IQ was estimated dividing MA by the child's chronological age (CA): MA/CA × 100. For this study, we have considered the non–verbal IQ scores (performance IQ). Sixty-two patients were not evaluable with standardized intelligence tests.

Males and females were represented in a different percentage in the total sample (83% vs. 17%; 820 males and 169 females) with a ratio between ASD males and ASD females similar to that reported in the literature (4.9:1). All cases of syndromic autism or with a known cause for ASD were excluded. No participant used psychotropic drugs in the last two months before the evaluation.

The current study was carried out according to the standards for good ethical practice and in accordance with the guidelines of the Declaration of Helsinki. Written informed consent from a parent/guardian of each participant was obtained when filling out the questionnaire.

### Measures

#### CBCL 1½−5

The Italian version of the Child Behavior Checklist (CBCL 1½−5) (64, 65) is one of the most widely used checklists consisting of 100 statements about the child's behaviors. The parents are asked to rate the frequency of each behavior on a three–point Likert scale (0, not true; 1, somewhat or sometimes true; 2, very true or often true). The CBCL provides seven syndrome scales scores (i.e., Emotionally Reactive, Anxious/Depressed, Somatic Complaints, Withdrawn, Aggressive Behavior, Attention Problems and Sleep Problems) and three summary scales scores (i.e., Internalizing, Externalizing and Total Problems). A T–score between 60 and 63 for summary scales, and a T–score between 65 and 69 for syndrome scales is considered in the borderline or clinical range. For this study we have in particular considered the DSM–Oriented scales (DOS): Affective Problems (AP), Anxiety Problems (AXP), Attention Deficit Hyperactivity Disorder Problems (ADHD), and Oppositional Defiant Problems (OP); for these four scales a T–score between 65 and 69 (borderline range) or above 70 is indicative for a clinically significant score. The items composing the syndromic scales and the DOS of the CBCL are unique confronting them to each other and also independent from the Pervasive Developmental Problems (PDP) scale.

In this study, we adopted the borderline clinical elevation cut–off score (T score ≥ 60 for summary scales and T score ≥ 65 for DOS), according to previous studies on screening (66–68) and comorbidity (16, 51) in young children with ASD.

### Procedure

All participants received a clinical diagnosis of ASD, were assessed with ADOS and were evaluated with psychometric tests when it was possible. Parents completed the CBCL at the beginning of the diagnostic process based on the behavior of their child in the last 6 months. For this study, the CBCL completed by mothers were preferred; when this was not possible, the CBCL was completed by fathers or by another close caregiver. Firstly, we examined the whole sample comparing different groups identified on the basis of single or multiple PC. Then, we looked for gender differences in PC. We also examined PC dividing the whole group on the base of higher or lower autistic behaviors measured by the PDP DOS on the CBCL.

### Data Analysis

All the continuous variables were examined for normality using skewness tests and Kolgomorov–Smirnov testing. The descriptive analyses, chi–square analysis and *t*–test were used for categorical and continuous independent variables, respectively. One–way analysis of variance (ANOVA) with Scheffe *post–hoc* test for multiple comparisons was performed to evaluate differences in age and CBCL scales among all groups. Analysis of covariance (ANCOVA) with Bonferroni *post–hoc* test for multiple comparisons was used to assess differences among the groups on CBCL scales, controlling for gender and age. In order to evaluate effect size, we measured: Cohen's d (d) for independent sample *t*–test, eta squared (η^2^ that represent the variance accounted for) for analysis of Variance, and Phi (ϕ) for non–parametric statistics (Chi–square). In order to evaluate the effect of PC on the severity of autism, we compared the group composed by ASD children without PC with different groups characterized by the presence of PC (Mono– or Multi–Comorbidity). To understand in which way each single type of PC (Affective Problems, Anxiety Problems, ADHD, Oppositive Problems) could specifically be associated with ASD level or IQ we compared the ADOS scores and the IQ scores among the ASD–only group and groups with a specific PC (mono–comorbid or multi–comorbid).

## Results

37.8% (374 participants) of our sample had a score over the borderline cutoffs on one or more of the DOS of the CBCL. It means that these participants had at least one PC in addition to ASD. In order to evaluate the relationship of PC with the severity of autism and cognitive level, we compared this PC group with the group of ASD children without PC (615 participants; 62.2% of the whole group). One–way ANCOVA (controlling for age and gender) revealed significantly higher mean scores on CSS–AS (*p* = 0.003) and on CSS–Total (*p* = 0.02) score in the PC group vs. the ASD–only group. Significantly higher performance IQ mean scores were present in the ASD–only group (*p* ≤ 0.0001) compared to the PC group (Table 2).

**Table 2:** ANCOVA (controlling for gender and age) on ADOS and Performance IQ between ASD with or without Psychiatric Comorbidity.

	**ASD–only (*N* = 615)**	**ASD+Psychiatric comorbidity (*N* = 374)**	**ANCOVA**		
			***F*–values**	***p*–value**	**Effect size**
ADOS CSS—Social Affect	**5.98 (1.95)**	**6.33 (1.95)**	**9.12** **(1,985)**	**0.003**	η^2^ = 0.01
ADOS CSS—RRB	6.93 (2.09)	6.98 (2.06)	0.73 (1,985)	0.40	–
ADOS CSS—Total score	**6.17 (1.95)**	**6.40 (1.95)**	**5.05** **(1,985)**	**0.02**	η^2^ = 0.003
Performance IQ	**81.20 (22.91)**	**75.93 (23.16)**	**13.15 (1,939)**	** < 0.0001**	η^2^ = 0.014

*Significant comparison are highlighted in bold*.

**Figure 1:**
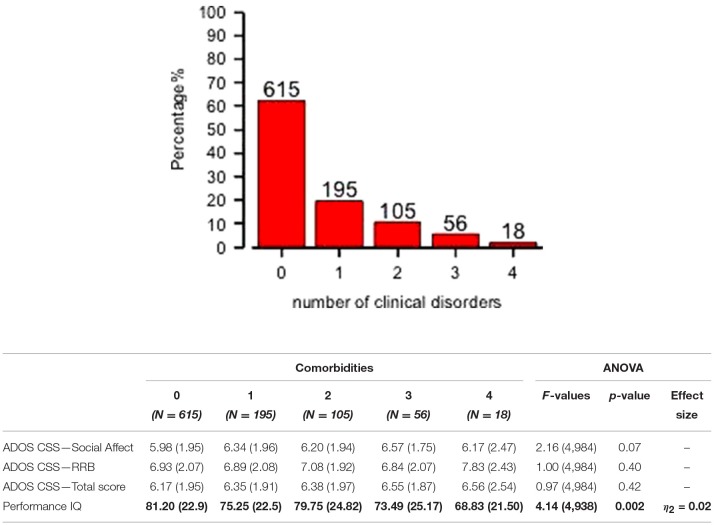
Distribution of mono– and multi–comorbidity across the whole sample and clinical comparisons. One–way ANOVA on ADOS scores and Performance IQ (means and SD) for all comorbidities groups are reported. Significant comparison are highlighted in bold.

Preschoolers presented a full range of different types of DOS (Table 1): Affective Problems were over the borderline cutoff in 23.4% of the whole sample; ADHD Problems in 17.3%; Anxiety Problems in 16.7%; Oppositional Problems in 7.9%. They were isolated (Mono–comorbid group) in 195 participants (52.1% of the 374 PC group; 19.7% of whole sample); 105 participants (28.1% of the 374 PC group; 10.6% of whole sample) had two types of PC; 56 participants (15% of the 374 PC group; 5.7% of whole sample) had three types of PC; 18 participants (4.8% of the 374 PC group; 1.8% of whole sample) were over the cutoff on all four DOS (Figure 1). One–way ANOVA revealed that participants with one (*n* = 195) or more PC (*n* = 179) have no statistically significant differences as far as IQ and ADOS scores are regarded. However, Figure 1 shows that participants who have four PC have also a significantly lower pIQ score (*p* = 0.002, η^2^ = 0.02). *Post–hoc* analyses using the Scheffe *post–hoc* criterion for significance indicated that the average IQ is significantly higher in the no PC group (*M* = 81.20, *SD* = 22.9) than in the group with at least one PC (*M* = 75.25, *SD* = 22.5, *p* = 0.02).

### Affective Problems

Two hundred and thirty-one participants had scores over the borderline cutoff on the Affective Problems DOS (Table 3). We found that this AP group (which has positive clinically significant scores in AP not taking in account mono– or multi–comorbidity) compared to participants without any borderline/clinical score on DOS (ASD–only group), had Higher CSS–AS (*p* = 0.001, *d* = 0.25) and CSS–Total Score (*p* = 0.01, *d* = 0.11), and lower pIQ (*p* = 0.001, *d* = 0.27). The AP group is composed of 81 participants with an isolated PC (mono–comorbid group) and of 150 multi–comorbid participants. ANCOVA with a Bonferroni *post hoc* test revealed significantly higher scores on CSS–SA (*p* = 0.004, η^2^ = 0.017) and CSS–Total (*p* = 0.01, η^2^ = 0.01) and significant lower score on pIQ (*p* = 0.002, η^2^ = 0.02) when the ASD–only group is compared to the mono–comorbid group; these differences were not found for multi–comorbid group. No significant difference was found for CSS–RRB scores within the groups identified.

**Table 3:** Clinical characteristics of ASD subjects grouped on the base of specific PC.

					**a**	**b**	**c**			
**AP**	**AP+ (*****N*** **= 231)**	**ASD–only (*****N*** **= 615)**	**t**	***p (ES)***	**Mono–Comorbidity (*****N*** **= 81)**	**Multi–Comorbidity (*****N*** **= 150)**	**ASD–only (*****N*** **= 615)**	**F**	***p–value***	**Effect Size**
CSS–SA	6.48 (1.97)	5.98 (1.95)	**3.25**	**0.001 (*****d*** **= 0.25)**	6.68 (1.88)	6.37 (2.02)	5.98 (1.95)	**6.66 (2,841)**	**0.001 a>c**	η2 = 0.017
CSS–RRB	7.17 (1.97)	6.93 (2.09)	1.54	0.12 (–)	7.17 (1.79)	7.17 (2.06)	6.93 (2.09)	1.57 (2,841)	0.19 ns	–
CSS–Total score	6.56 (1.95)	6.17 (1.95)	**2.51**	**0.01 (*****d*** **= 0.11)**	6.65 (1.68)	6.51 (2.08)	6.17 (1.95)	**4.19 (2,841)**	**0.01 a>c**	η2 = 0.01
Performance IQ	74.87 (23.48)	81.20 (22.91)	**−3.46**	**0.001 (*****d*** **= 0.27)**	72.96 (20.26)	75.93 (25.05)	81.20 (22.91)	**7.09 (2,801)**	**0.001 c>a c>b**	η2 = 0.02
**ADHD**	**ADHD+ (*****N*** **= 171)**	**ASD–only (*****N*** **= 615)**	**t**	***p***	**Mono– Comorbidity (*****N*** **= 62)**	**Multi– Comorbidity (*****N*** **= 109)**	**ASD–only (*****N*** **= 615)**			
CSS–SA	6.15 (1.87)	5.98 (1.95)	**2.05**	**0.04 (*****d*** **= 0.09)**	6.21 (1.90)	6.39 (1.86)	5.98 (1.95)	2.74 (2,781)	0.06 ns	–
CSS–RRB	6.91 (2.19)	6.93 (2.09)	−0.78	0.43 (–)	6.42 (2.30)	6.99 (2.11)	6.93 (2.09)	1.86 (2,781)	0.13 ns	–
CSS–Total score	6.61 (1.95)	6.17 (1.95)	1.15	0.24 (–)	6.15 (2.10)	6.49 (1.86)	6.17 (1.95)	2.05 (2,781)	0.10 ns	–
Performance IQ	75.05 (25.24)	81.20 (22.91)	**−2.77**	**0.006 (*****d*** **= 0.25)**	76.88 (24.67)	74.57 (25.63)	81.20 (22.91)	**4.58** (2,743)	**0.003 c>b**	η2 = 0.012
**OP**	**OP+ (*****N*** **= 80)**	**ASD–only (*****N*** **= 615)**	**t**	***p***	**Mono– Comorbidity (*****N*** **= 9)**	**Multi– Comorbidity (*****N*** **= 71)**	**ASD–only (*****N*** **= 615)**			
CSS–SA	6.42 (1.99)	5.98 (1.95)	1.86	0.06 (–)	6.33 (2.73)	6.43 (1.89)	5.98 (1.95)	**3.13** (2,690)	**0.02 b>c**	η2 = 0.006
CSS–RRB	7.13 (2.07)	6.93 (2.09)	0.80	0.42 (–)	7.78 (1.71)	7.04 (2.11)	6.93 (2.09)	0.74 (2,690)	0.52 ns	–
CSS–Total score	6.56 (2.13)	6.17 (1.95)	1.66	0.09 (–)	6.89 (2.80)	6.52 (2.05)	6.17 (1.95)	1.98 (2,690)	0.11 ns	–
Performance IQ	70.55 (22.48)	81.20 (22.91)	**−3.79**	**0.0001 (*****d*** **= 0.46)**	68.33 (22.82)	70.85 (22.60)	81.20 (22.91)	**6.17** (2,660)	** < 0.001 c>a**	η2 = 0.03

*Each specific PC is compared to ASD–only subjects without PC. AP, Affective Problems; ADHD, Attention Deficit/Hyperactivity problems; OP, Oppositional Problems. Significant comparison after Bonferroni correction are highlighted in bold*.

### Anxiety Problems

One hundred sixty-five participants had scores over the borderline cutoff on the Anxiety Problems DOS; this sample was composed of 43 children with mono–comorbidity (positive only on AXP scale) and of 122 multi–comorbid children (positive to AXP scale and other DOS). ANCOVA revealed no significant difference on CSS–SA scores, CSS–RRB scores, CSS–Total scores and pIQ between AXP and ASD–only or for mono– and multi–comorbid AXP children.

### ADHD Problems Cluster

One hundred seventy-one children had scores over the borderline cutoff on the ADHD problems of the DOS (Table 3). Differences between this group (mono–comorbid and multi–comorbid) and the ASD–only group showed that ADHD group was associated with Higher CSS–SA (*p* = 0.04, *d* = 0.09) and lower IQ (*p* = 0.006, *d* = 0.25) compared with the ASD–only children. The ADHD sample was composed of 62 participants with an isolated PC (positive only on ADHD scale) and 109 multi–comorbid participants (positive to ADHD scale and other DOS). ANCOVA failed to reveal significant differences on all CSS scores among groups, but multi–comorbid ADHD group showed lower statistically significant scores on pIQ (*p* = 0.003, η^2^ = 0.012) compared to the ASD–only group.

### Oppositional Problems

Eighty children showed scores over the borderline cutoff on the Oppositional Problems DOS (Table 3). This group, composed of mono–comorbid and multi–comorbid participants, showed lower pIQ (*p* < 0.0001, *d* = 0.46) compared to the group of children without any comorbidity (ASD–only group). This OP sample is composed of 9 children with an isolated PC (mono–comorbid group) and 71 multi–comorbid children (positive to OP scale and to other DOS). ANCOVA with a Bonferroni *post hoc* test revealed a significantly higher score on CSS–SA (*p* = 0.02, η^2^ = 0.006) and lower score on pIQ (*p* = 0.002, η^2^ = 0.03) when mono–comorbid group is compared to the ASD–only group. In addition, by comparing CSS–RRB scores between groups we found a significant interaction between group and age [*F*_(2, 689)_ = 4.03 *p* = 0.02, η^2^ = 0.01].

Moreover, we compared groups based on the type of psychiatric mono–comorbidity and ANCOVA was not able to detect differences on the clinical variables considered (Table 4). In order to evaluate the effect of having different clusters of multi–comorbidity we compared groups composed of any type of different clustering (i.e., AP and AXP, AP, and ADHD, ADHD and AXP, AP, and AXP and OP, etc.); also in this case different ANCOVA did not reveal any significant differences among groups on the considered clinical variable.

**Table 4:** Clinical differences among group of ASD subjects with specific psychiatric mono–comorbidity.

	**DSM–IV oriented scales**				**ANCOVA**		
	**AP+ *(N* = *81)***	**AXP+ *(N* = *43*)**	**ADHD+ *(N* = *62)***	**OP+ *(N* = *9)***	***F*–values**	***p–value***	**Effect size**
CSS–SA	6.58 (1.83)	5.91 (2.01)	6.11 (1.81)	6.33 (2.73)	1.49 (3,189)	0.21	–
CSS–RRB	7.18 (1.74)	6.84 (2.24)	6.30 (2.33)	7.78 (1.71)	1.86 (3,189)	0.13	–
CSS–Total score	6.58 (1.65)	5.98 (1.75)	6.02 (2.04)	6.89 (2.80)	1.53 (3,189)	0.20	–
Performance IQ	72.93 (20.26)	78.70 (23.57)	76.88 (24.67)	68.33 (22.82)	0.92 (3,182)	0.42	–

Finally, we have considered the distribution of PC taking into account the severity of autism and gender differences. In order to investigate the effect of the severity of autism, we have divided the whole group on the basis of having a PDP score over (PDP+; 682 participants) or under (PDP–; *n* = 307) the borderline cut–off on this scale. The PDP+ group show significantly higher mean scores on all DOS and higher number of participants with PC compared to the group of ASD preschoolers with the score under the borderline cut–off (Table 5).

**Table 5:** Clinical comparison of ASD subjects with high scores on CBCL–PDP scale and subjects with low score on CBCL–PDP scale.

		**PDP high (*n* = 682) M (*SD*)**	**PDP low (*n* = 307) M (*SD*)**	***t–*test**	***p–value***	**Effect Size**
Age (months)		44.00 (13.40)	44.05 (14.53)	−0.05	0.95	***–***
Performance IQ		76.68 (23.33)	84.82 (22.83)	**−5.02**	**<0.001**	*d* = 0.35
CSS–SA		6.36 (1.87)	5.57 (2.04)	**5.98**	**<0.001**	*d* = 0.40
CSS–RRB		7.09 (2.00)	6.64 (2.21)	**3.13**	**<0.001**	*d* = 0.21
CSS– Total score		6.49 (1.87)	5.73 (2.02)	**5.72**	**<0.001**	*d* = 0.39
Pervasive developmental problems		73.88 (6.21)	57.2 (4.6)	**42.43**	**<0.0001**	*d* = 3.05
**Psychiatric Comorbidity**				***t*****–test** **or** ***X**^**∧**^**2***	***p–value***	**Effect Size**
Affective problems	*A*	60.94 (9.06)	54.12 (5.84)	**12.09**	**<0.001**	*d* = 0.89
	*B*	30.6	7.2	**65.19**	**<0.0001**	ϕ = 0.25
Anxiety problems	*A*	58.61 (8.70)	52.59 (4.44)	**11.45**	**<0.0001**	*d* = 0.87
	*B*	22.7	3.3	**57.73**	**<0.0001**	ϕ = 0.24
Attention deficit/Hyperactivity problems	*A*	59.22 (7.04)	54.65 (5.67)	**9.98**	**<0.0001**	*d* = 0.71
	*B*	22.6	5.5	**43.11**	**<0.0001**	ϕ = 0.20
Oppositional problems	*A*	55.74 (6.62)	51.81 (3.43)	**9.82**	**<0.0001**	*d* = 0.74
	*B*	11.1	0.7	**32.08**	**<0.0001**	ϕ = 0.18

*A, mean and SD; B, number of subjects in the borderline/clinical range expressed as percentage of subjects with PDP high or low score*.

*Significant comparison are highlighted in bold*.

Regarding gender differences, we found a statistically significant difference on RRB–CSS where males show a higher score when compared to females with ASD. No significant differences were found for age, pIQ, CSS Total score and CSS–SA. Males and females did not show any difference on mean DOS score over the borderline cut-off (Table 6).

**Table 6:** Gender differences: comparison of clinical characteristics and of PC in male vs. female.

		**Male M (*SD*)**	**Female M (*SD*)**	***t–*test**	***p–value***	**Effect size**
Age		44.08 (13.92)	43.67 (13.06)	0.32	0.74	–
Performance IQ		76.68 (23.17)	76.58 (23.82)	1.61	0.10	–
CSS–SA		6.11 (02.16)	6.15 (1.92)	−0.20	0.84	–
CSS–RRB		7.02 (2.04)	6.57 (2.16)	**2.64**	**0.008**	*d* = 0.21
CSS– Total score		6.27 (1.92)	6.20 (2.01)	0.48	0.62	–
Pervasive developmental problems		68.89 (9.80)	67.44 (9.15)	1.75	0.08	–
**Psychiatric comorbidity**				***t*****–test** **or** ***X**^**∧**^**2***	***p–value***	**Effect size**
Affective problems	*A*	58.95 (8.86)	58.21 (8.36)	0.99	0.32	–
	*B*	23.5	22.5	0.08	0.76	–
Anxiety problems	*A*	56.98 (8.30)	55.97 (7.13)	**2.06**	**0.04**	*d* = 0.09
	*B*	17.6	12.4	2.65	0.10	–
Attention deficit/ Hyperactivity problems	*A*	57.75 (6.89)	58.05 (7.33)	−0.50	0.61	–
	*B*	17.0	18.9	0.37	0.53	–
Oppositional problems	*A*	54.50 (6.11)	54.59 (6.02)	−0.14	0.88	–
	*B*	7.9	7.7	0.01	0.91	–

*A, mean and SD; B, number of subjects in the borderline/clinical range expressed as percentage of male or female. Significant comparison are highlighted in bold*.

Finally, in order to study possible differences between younger (≤36 months) and older (>36 months) subjects on ASD symptom severity and PC, the sample was dichotomized on the basis of age in 333 children younger or equal than 36 months (33% of sample) and 656 older than 36 months (67% of sample). The comparison between the two groups revealed higher scores for the younger groups on CSS–SA [*t*_(987)_ = 3.58, *p* < 0.0001, *d* = 0.23], CSS–RRB [*t*_(987)_ = 2.75, *p* = 0.006, *d* = 0.17], and CSS–Total scores [*t*_(987)_ = 4.22 *p* < 0.0001, *d* = 0.27], but no difference as far as prevalence of PC is regarded (Table 7).

**Table 7:** Clinical differences between younger (≤36 months) and older (>36 months) subjects.

		**≤36 m (*n* = 333) M (*SD*)**	**>36 m (*n* = 656) M (*SD*)**	***t*–test**	***p–value***	**Effect size**
Age (months)		29.30 (4.90)	51.48 (10.36)	−36.97	< 0.0001	*d* = 2.73
Performance IQ		78.94 (22.11)	79.34 (23.88)	−0.24	0.84	–
CSS–SA		**6.43 (2.07)**	**5.96 (1.88)**	**3.58**	** < 0.0001**	*d* = 0.23
CSS–RRB		**7.20 (2.24)**	**6.82 (1.98)**	**2.75**	**0.006**	*d* = 0.17
CSS– Total score		**6.62 (2.18)**	**6.07 (1.80)**	**4.22**	** < 0.0001**	*d* = 0.27
Pervasive developmental problems		68.29 (9.90)	68.82 (9.61)	−0.81	0.41	–
**Psychiatric comorbidity**				***t*****–test or** ***X**^**∧**^**2***	***p–value***	**Effect size**
Affective problems	*A*	58.69 (9.08)	58.89 (8.63)	−0.32	0.74	–
	*B*	22.9	24.3	1.08	0.60	–
Anxiety problems	*A*	**55.85 (7.69)**	**57.20 (8.31)**	**−2.47**	**0.01**	*d* = 0.16
	*B*	17.7	14.7	1.40	0.23	–
Attention deficit/Hyperactivity problems	*A*	57.21 (6.83)	58.10 (7.03)	−1.91	0.06	–
	*B*	18.8	14.4	2.93	0.08	–
Oppositional problems	*A*	54.24 (6.20)	54.66 (6.04)	−1.00	0.31	–
	*B*	8.2	7.2	0.31	0.57	–

*A, mean and SD; B, number of subjects in the borderline/clinical range expressed as percentage). Significant comparison are highlighted in bold*.

## Discussion

This study aims to explore psychiatric comorbidity in a wide sample of ASD preschoolers, searching for the impact of gender, symptom severity and intelligence on PC. For this purpose, we have used the DSM Oriented Scales (DOM) of the CBCL 1½−5 that have proven validity to identify PC in ASD preschoolers (51). Nevertheless, their use is sparse in literature and, to our knowledge, this is the first time that they are applied in a very large sample of preschoolers with ASD.

Results revealed that 37.8% of the participants had at least one PC in addition to ASD. This finding is not surprising since significant genetic overlap between the diverse group of neurodevelopmental disorders—for instance ASD and ADHD—(69) and between different psychiatric diseases—e.g., ASD and depression—has been identified (70). The relative low rate of PC in our sample can be interpreted from a developmental perspective. Specifically, it is plausible that in the toddler–age the majority of the child's emotional and behavioral problems could be explained by a diagnosis of ASD, whereas, with increasing age and consequently social, adaptive and cognitive demands, new internalizing and externalizing disorders emerge. Accordingly, 71% of children (21), 74% of adolescents (23), and 73% of adults with ASD (8) have been described as affected by at least one other psychiatric diagnosis.

Our results indicated that children with ASD combined with one or more PC had higher severity of autism symptoms and lower IQs than ASD children without PC, in contrast to previous findings where no relationship with these clinical features was found (21, 71), even taking in account the effect of gender and age, according to this we found a small to medium (η^2^ = 0.014 to 0.02) effect size in our analysis. Of particular importance is the detection of PC in individuals with ASD plus intellectual disability in order to avoid the diagnostic overshadowing (14) that is the attribution of all symptoms to intellectual impairment instead of to specific PC.

Moreover, 18.1% of our whole sample had two or more PC associated with ASD (multi–comorbid group). This percentage is lower than that reported by Simonoff et al. (21); in this study 52% of participants had multiple PC and 38% had three or more PC, while in our sample 10.6% participants had two types of PC, 5.7% participants had three types of PC, and only 1.8% satisfy all the four PC we considered. Furthermore, the cumulative percentage of our children with at least two PC in addition to autism was significantly lower (18.2 vs. 50% approximately) than that reported in another study (9). The lower average age of our sample as well as the exclusion of other types of psychiatric disorders not detected by the CBCL (e.g., obsessive compulsive disorder, specific phobias) could partly justify this finding. However, some behaviors of ASD children could be misinterpret by clinicians or parents; difficulties in disentangling symptoms of PC from ASD symptoms (i.e., withdrawn) could create an under–estimation—but also an over–estimation—of PC in ASD subjects depending on setting or on the training of the professionals (72).

As far as the specific PC, we confirm the considerable additional presence of affective problems, ADHD, and anxiety problems among our participants, as previously highlighted by other studies (73, 74).

The overall rate of affective problems that we found (23.4%) is similar to the one reported by Leyfer et al. (9) in a sample of 9–years–old ASD subjects, using a modified version of the K–SADS. Conversely, Salazar et al. (27) detected that only 14.6% of their preschool and elementary–school aged children met criteria for major depressive disorder, using the Preschool Age Psychiatric Assessment (PAPA) interview (39). A significant lower rate of major depression and dysthymic disorder (1.4%) was observed by Simonoff et al. (21) in a sample of ASD children and adolescents aged 10–14 years, using the Child and Adolescent Psychiatric Assessment–parent version (CAPA) (75). The fact that in our sample the affective symptoms have been evaluated through the CBCL may have had an impact on results. In fact, it is worthy of note that four out ten items that make up the Affective Problems (AP) scale of the CBCL 1 ½−5 (i.e., 24: doesn't eat well; 38: has trouble getting to sleep; 49: overeating; 74: sleeps less than most kids during day and/or night) are strictly related to neurovegetative symptoms, such as sleeping and eating problems: these features, as well as being part of clinical depression are also disturbances that occurred at a higher rate in ASD individuals than in typically developing (TD) children, independently from the associated PC (76, 77). For example, as far as sleep problems, we found that 56% of our sample had at least one sleep problem (i.e., scored 2 in at least one out of the seven sleep items in the CBCL) and this percentage is close to 53% found by Krakowiak et al. (78) who included in their “sleep problems group” children with ASD and at least one frequent sleep problem. Therefore, it is possible that the inclusion of eating and sleep problems in the CBCL–AP scale has led to overestimate the rate of affective problems in preschoolers with ASD. Similarly, other AP items, such as the 43 (“looks unhappy without good reason”), the 89 (“underactive, slow moving, or lacks energy”), and the 71 (“shows little interest in things around him/her”) could be part of the ASD early presentation in which troubles of affect are frequently reported (79), besides being depressive symptoms. However, previous investigation supports the use of the CBCL 1.5–5 to assess for emotional disorders in preschoolers with ASD (80) and replicated studies have demonstrated the construct validity of the CBCL for evaluating PC in older ASD subjects (29, 30); in particular the AP scale, despite including sleep and eating problems, showed a statistically significant correlation with Depression based on the K–SADS (81). More broadly, the association between AP and ASD should be interpreted with caution, since a considerable phenotypic overlap between these two conditions exists (82): consequently, the accurate diagnosis of depression in toddlers with ASD remains a challenge. Further, our results show that children with Affective Problems had notable association with lower pIQ and more severe autism, in both case we found a small to medium effect size. Previous studies on the relationship between intellectual disability and affective comorbidities in young individuals with ASD have been inconsistent. Some authors fail to find a relationship between intellectual disability and depression in subjects with ASD (83, 84), while others identified a decreased risk of depression in children with ASD and intellectual disability (27, 85, 86). Thus, our clear results of an quite strong association between the presence of AP and a more severe autism with lower pIQ set the stage for a more careful consideration of the relationship among intellectual disability, affective problems and autism.

We detected that 17.3% of participants exceed the cutoff in the ADHD scale, a percentage lower than that observed in previous research (9, 10, 15, 21, 27). Also for this PC our lower percentage may be partly explained by the lower ages in our sample. In fact, symptoms of ADHD may emerge in toddlerhood (87), but generally increase with age: for instance, in a clinically referred sample of children with ASD, 40% of 3–5-year old and over 50% of 6–12-year–old children met DSM-IV criteria for ADHD (88). Nevertheless, our percentages of children with ADHD are only slightly lower than that detected using the CBCL in a recent investigation (51) where it was reported that 22% of their preschoolers had ADHD. Thus, it is possible to suggest that our lower percentage is due to the ADHD–DOS which is more conservative than other instruments to individuate ADHD. Our results show significantly lower pIQ in ASD comorbid with ADHD, with an effect size ranging between small and medium magnitude; while some investigations suggest that rates of comorbid ADHD are high regardless the level of IQ (85, 89), others reported a more severe ASD phenotype when associated with ADHD, not only in terms of lower IQ, but also of higher autistic symptoms and more behavioral problems (90, 91). Our results support these latter findings, since the ASD plus ADHD children had significant lower pIQ as well as considerable rate of multi–comorbidity; this finding indirectly supports the evidence of a specific phenotype characterized by ASD plus ADHD which may increase the risk of further comorbidity (92).

Also the percentage of children affected by anxiety problems (16.7% of the whole sample) is lower than that observed in other researches (9, 21, 27, 92) and meta–analysis (93). The lower ages of our large sample could partly explain this finding: accordingly, a cross–sectional recent study compared the levels of anxiety in different age–ranges and found an increase of anxiety levels from toddlerhood to childhood (94). Similarly, different studies detected a positive association between anxiety levels and chronological age in toddlers (95), children (27) and adolescents with ASD (96). However, higher rates of anxiety problems were detected also in ASD samples with age similar to ours: for example, Llanes et al. (51), using the CBCL in their subgroup of preschoolers, identify an anxiety problem in 31% of the sample that doubles up the percentage in our sample. Crucially, all the enrolled subjects in that research had an IQ on the WPPSI–III of 50 or above, and the mean IQ score of the participants was within average levels, while in our sample also subjects with a pIQ lower than 50 were included. This difference on IQ scores could be the second reason for the lower percentages of AXP, since literature frequently reported that higher levels of anxiety are associated with better cognitive skills (97, 98). A third factor, related to the impairment in receptive and expressive language skills (94), could be responsible for the low prevalence of anxiety problems in our ASD sample: in fact, we could suppose that the low mean chronological age and the below–average cognitive level impacted on language and consequently on their ability to express anxious symptoms.

A relative small percentage of our subjects, 7.9% of total sample, exceed the cutoff for Oppositional Defiant Problems: this rate is not significantly different from the prevalence estimates of 7% (99) and 10% (40) for preschoolers in the general population. In previous studies higher rates of OP have been reported – 37% in de Bruin et al. (22); 13% in Gadow et al. (100); 30% in Simonoff et al. (21)–and these symptoms seem to increase over time: in fact, samples composed of older ASD children (85, 88, 101) exhibited a more elevated prevalence of these behavioral problems. It was suggested that the increase in social stressors (e.g., academic and peer demands) could have a role in this behavioral modification with age of ASD individuals. Our results show also that OP symptoms are more likely to be present in ASD preschoolers with lower intellective functioning, in accordance with some (101, 102), but not all literature (85, 103). Secondly, it is important to consider that some behavior of ASD children can be interpreted as oppositional by parents (for instance the items “defiant,” “disobedient,” “stubborn,” “uncooperative”) instead of the consequence of the poor attention to social stimuli and/or impairment in social understanding e.g., (104–106) typical of ASD rather than symptoms of a real OP.

It is important to highlight that 31% of our participants does not reach the borderline scores on the PDP scale. Even if the PDP scale showed an high accuracy in distinguishing preschoolers with ASD from peers with typical development (66) and from peers with other psychiatric disorders (67), the sensitivity of this scale to detect ASD subjects is lower than the sensitivity of other CBCL scales to detect the corresponding PC (72, 107). Subjects positive to the PDP scale are, as expected, more impaired in terms of ADOS severity and intellective functioning, but, interestingly, they are also characterized by more frequent PC as highlighted by significantly higher mean scores on all DOS in comparison to subjects below the cut–off score at the PDP scale, with a moderate to strong effect size (all *d* > 0.70), suggesting a significant effect of having borderline scores on PDP scale. Therefore, in accordance with previous studies (108, 109), we could speculate that the PDP scale could be used as a measure not only of the possible presence of an ASD disorder but also of likely different functional impairments.

The comparison of the DOS scores between males and females participants did not reveal any statistically significant difference as far as scores within the borderline/clinical range of these scales are concerned. Some previous studies suggest a different phenotype in terms of PC in female than in male children with ASD (27, 47, 50, 110, 111), whereas other investigations failed to find clear gender differences (112, 113) or gender differences that reflect those found in typical young children (114). Therefore, data are still limited and inconclusive on this theme and further research is needed on this under–explored issue.

Interestingly, the comparison between younger (≤36 months) and older (>36 months) patients of our sample highlighted significantly higher symptoms severity in the younger group, with a small to moderate effect size (all *d* > 0.10). This result is in line with previous investigations in which the severity of ASD symptoms was negatively correlated with age at first ASD diagnosis (115, 116). Nevertheless, the more severe ASD symptoms at an earlier age is not linked to a more severe PC, which seem stable across ages and not influenced by autism *per se*.

## Conclusions

Our findings should be considered in light of some methodological limitations. First, it is important to highlight that, in order to receive one or more specific DSM comorbid diagnoses, ASD patients can be in–depth evaluated by trained clinicians with expertise in childhood psychiatric disorders. In fact, we relied only on the use of the CBCL 1.5–5 for the evaluation of PC in young children with ASD, which has however shown a good ability to assess for emotional and behavioral disorders in preschoolers with ASD (80). The absence of a subsequent clinical evaluation to confirm a diagnosis of PC may have caused a certain percentage of false positives. On the other hand, the presence of some false negatives cases should be considered. Since the preschoolers of the current study are referred for a diagnostic evaluation to three tertiary centers specific for ASD, it is possible that parents are more focused on ASD–Specific concerns (e.g., communication/language delays, social deficits) than on non–ASD–Specific concerns (e.g., inattention and hyperactivity, eating/feeding, sleep difficulties, tantrums or inappropriate behaviors) (117).

More broadly, parent ratings inevitably involve the risk of several parental bias, including difficulty in interpreting the questions and quantifying the behaviors, reluctance to acknowledge the child's problems, and lack of motivation to complete the instrument accurately. However, literature indicates that parents are generally reliable informants about the behavioral and emotional problems of their child (118), aside from providing valuable and unique information about the child's behaviors in the home environment and in specific situations (e.g., eating and sleeping habits). The lack of parents' history of psychiatric disorders is another limitation of this study. This information is important not only to increase comprehension of PC in ASD children Wiggins et al. (119), but also to accurately interpreting parent–report. For example, the possible negative bias of anxious or depressed parents can lead them to overestimate the amount of symptoms of their own kid (120). On the other hand, parents with externalizing psychopathology, but without insight into their own condition, could underestimate this type of symptoms in their child, considering them as part of a typical behavior.

The cross–sectional design of this study precluded us to draw inferences about the stability of CBCL profiles and their impact on the developmental trajectories of preschoolers with ASD. The few longitudinal studies on this topic detected a low/declining trajectory for internalizing problems in 70% of the sample (50), or an association between low scores on ADHD related traits over time and positive outcome (121). Future longitudinal investigations are therefore necessary and could also help to clarify whether the treatment of PC had a positive impact on adaptive function and core features of ASD patients. Moreover, we do not have a longitudinal evaluation of subjects diagnosed before or at 36 months of chronological age confirming the clinical diagnosis of ASD. However, diagnoses of ASD in toddlers have been found to be accurate and stable across time in studies of high–risk siblings (122), community–based settings (123) and clinic–referred samples (124). Moreover, the high symptoms severity in our younger ASD subjects supports the stability of their diagnosis, since the severity of ASD symptoms has been indicated as a factor contributing to the diagnostic stability of ASD (116).

Finally, the information about the sibling status could be of valuable relevance, since the presence of typical or atypical older siblings can impact on the parents' sensitivity to reliably rate symptoms of their younger child (125, 126).

In conclusion, this study suggests that in persons with ASD, PC occur early in life necessitating the need for their early detection that could improve our capacity for a more tailored intervention.

## Data Availability

The anonymized datasets generated for this study are available on request to the corresponding author.

## Ethics Statement

This study was carried out in accordance with the recommendations of IRCCS Stella Maris committee with written informed consent from all subjects. All subjects gave written informed consent in accordance with the Declaration of Helsinki. The protocol was approved by the IRCCS Stella Maris committee.

## Author Contributions

FM, MT, SV, MP, and SC participated in the design of the work and wrote the first draft of the manuscript. MT analyzed the data. MT, AN, GV, SG, FA, ES, and CL evaluated the patients and collected the data. SV, SG, SC, and CL helped to evaluate, edit the manuscript and performed critical revision. Each Author has seen and approved the submission of this version of the manuscript and takes full responsibility for the manuscript.

### Conflict of Interest Statement

The authors declare that the research was conducted in the absence of any commercial or financial relationships that could be construed as a potential conflict of interest.
